# Supplementary material for: Pyruvate Dehydrogenase Kinase 1 inhibition mediated oxidative phosphorylation enhancement in cartilage promotes osteoarthritis progression
Source: BMC Musculoskelet Disord. 2023 Jul 20;24:597. doi: 10.1186/s12891-023-06585-6 (PMC10357736; doi:10.1186/s12891-023-06585-6)
Supplement: Supplementary file 1 — Additional file 1. [file 12891_2023_6585_MOESM1_ESM.zip › KEGG.Gsea.1653622432464/KEGG_GALACTOSE_METABOLISM.html]

Details for gene set KEGG\_GALACTOSE\_METABOLISM[GSEA]

|  || Dataset | 55457\_collapsed\_to\_symbols.cls.cls #OA\_versus\_normal.cls.cls #OA\_versus\_normal\_repos |
| Phenotype | cls.cls#OA\_versus\_normal\_repos |
| Upregulated in class | normal |
| GeneSet | KEGG\_GALACTOSE\_METABOLISM |
| Enrichment Score (ES) | -0.16807242 |
| Normalized Enrichment Score (NES) | -0.5354201 |
| Nominal p-value | 0.9863946 |
| FDR q-value | 1.0 |
| FWER p-Value | 1.0 |
Table: GSEA Results Summary

  

Fig 1: Enrichment plot: KEGG\_GALACTOSE\_METABOLISM      
 Profile of the Running ES Score & Positions of GeneSet Members on the Rank Ordered List

  

| SYMBOL | TITLE | RANK IN GENE LIST | RANK METRIC SCORE | RUNNING ES | CORE ENRICHMENT || 1 | GLB1 | galactosidase beta 1 [Source:HGNC Symbol;Acc:HGNC:4298] | 337 | 0.731 | 0.0964 | Yes |
| 2 | GAA | alpha glucosidase [Source:HGNC Symbol;Acc:HGNC:4065] | 1149 | 0.486 | 0.1150 | Yes |
| 3 | GALT | galactose-1-phosphate uridylyltransferase [Source:HGNC Symbol;Acc:HGNC:4135] | 1666 | 0.404 | 0.1426 | Yes |
| 4 | PFKM | "phosphofructokinase, muscle [Source:HGNC Symbol;Acc:HGNC:8877]" | 2260 | 0.331 | 0.1521 | Yes |
| 5 | AKR1B1 | aldo-keto reductase family 1 member B [Source:HGNC Symbol;Acc:HGNC:381] | 3138 | 0.236 | 0.1237 | No |
| 6 | HK3 | hexokinase 3 [Source:HGNC Symbol;Acc:HGNC:4925] | 3792 | 0.178 | 0.1028 | No |
| 7 | PGM1 | phosphoglucomutase 1 [Source:HGNC Symbol;Acc:HGNC:8905] | 3990 | 0.157 | 0.1138 | No |
| 8 | GLA | galactosidase alpha [Source:HGNC Symbol;Acc:HGNC:4296] | 4172 | 0.140 | 0.1233 | No |
| 9 | GALK2 | galactokinase 2 [Source:HGNC Symbol;Acc:HGNC:4119] | 4945 | 0.076 | 0.0761 | No |
| 10 | G6PC1 | glucose-6-phosphatase catalytic subunit 1 [Source:HGNC Symbol;Acc:HGNC:4056] | 5389 | 0.041 | 0.0486 | No |
| 11 | PFKP | "phosphofructokinase, platelet [Source:HGNC Symbol;Acc:HGNC:8878]" | 5500 | 0.032 | 0.0455 | No |
| 12 | PFKL | "phosphofructokinase, liver type [Source:HGNC Symbol;Acc:HGNC:8876]" | 5632 | 0.021 | 0.0389 | No |
| 13 | GALK1 | galactokinase 1 [Source:HGNC Symbol;Acc:HGNC:4118] | 5785 | 0.009 | 0.0287 | No |
| 14 | HK1 | hexokinase 1 [Source:HGNC Symbol;Acc:HGNC:4922] | 6728 | -0.058 | -0.0348 | No |
| 15 | LALBA | lactalbumin alpha [Source:HGNC Symbol;Acc:HGNC:6480] | 7143 | -0.092 | -0.0515 | No |
| 16 | LCT | lactase [Source:HGNC Symbol;Acc:HGNC:6530] | 8444 | -0.189 | -0.1207 | No |
| 17 | UGP2 | UDP-glucose pyrophosphorylase 2 [Source:HGNC Symbol;Acc:HGNC:12527] | 8735 | -0.211 | -0.1077 | No |
| 18 | G6PC2 | glucose-6-phosphatase catalytic subunit 2 [Source:HGNC Symbol;Acc:HGNC:28906] | 9088 | -0.239 | -0.0950 | No |
| 19 | HK2 | hexokinase 2 [Source:HGNC Symbol;Acc:HGNC:4923] | 10030 | -0.325 | -0.1136 | No |
| 20 | MGAM | maltase-glucoamylase [Source:HGNC Symbol;Acc:HGNC:7043] | 10106 | -0.331 | -0.0639 | No |
| 21 | GCK | glucokinase [Source:HGNC Symbol;Acc:HGNC:4195] | 10137 | -0.335 | -0.0101 | No |
| 22 | B4GALT2 | "beta-1,4-galactosyltransferase 2 [Source:HGNC Symbol;Acc:HGNC:925]" | 10490 | -0.372 | 0.0250 | No |
| 23 | GALE | UDP-galactose-4-epimerase [Source:HGNC Symbol;Acc:HGNC:4116] | 11227 | -0.461 | 0.0451 | No |
| 24 | B4GALT1 | "beta-1,4-galactosyltransferase 1 [Source:HGNC Symbol;Acc:HGNC:924]" | 11530 | -0.505 | 0.1064 | No |
Table: GSEA details [plain text format]

  

Fig 2: KEGG\_GALACTOSE\_METABOLISM      
 Blue-Pink O' Gram in the Space of the Analyzed GeneSet

  

Fig 3: KEGG\_GALACTOSE\_METABOLISM: Random ES distribution      
 Gene set null distribution of ES for **KEGG\_GALACTOSE\_METABOLISM**
